# Interventional Radiology Approaches for Liver Metastases from Thyroid Cancer: A Case Series and Overview of the Literature

**DOI:** 10.1007/s12029-021-00646-6

**Published:** 2021-05-17

**Authors:** Alice Nervo, Alberto Ragni, Francesca Retta, Marco Calandri, Carlo Gazzera, Marco Gallo, Alessandro Piovesan, Emanuela Arvat

**Affiliations:** 1 Oncological Endocrinology Unit, Department of Medical Sciences, Città Della Salute E Della Scienza Hospital, University of Turin, Turin, Italy; 2 Diagnostic Imaging and Interventional Radiology Department, Città Della Salute E Della Scienza Hospital, University of Turin, Turin, Italy; 3 Endocrinology and Metabolic Diseases Unit, AO SS. Antonio E Biagio E Cesare Arrigo Hospital, Alessandria, Italy

**Keywords:** Thyroid carcinoma, Loco-regional treatment, Hepatic metastases, Radiofrequency, Chemoembolization

## Abstract

**Background:**

Liver metastases (LMs) from thyroid cancer (TC) are relatively uncommon in clinical practice and their management is challenging. Interventional radiology loco-regional treatments (LRTs), including radiofrequency ablation (RFA) and trans-arterial chemoembolization (TACE), have been successfully employed to treat LMs from various types of cancer.

**Methods:**

We analyzed the role of LRTs in the management of unresectable LMs from differentiated and medullary TCs performed at our institution from 2015 to 2020. A review of the available English literature regarding this topic was also performed.

**Results:**

Six hepatic LRTs were performed in 4 TC patients with LMs, in 2 cases after the start of treatment with a tyrosine kinase inhibitor (TKI). A partial response was obtained in 2 patients; the diameter of the largest targeted lesion was 18 mm in both of them. The remaining procedures were performed on larger lesions and a stable disease was achieved in all but one case. Acute LRT-related complications were transient and mild. In literature, the largest studies were focused on TACE in LMs from MTC, showing good tolerance and remarkable disease control, especially in case of limited liver tumour involvement.

**Conclusion:**

LRTs for LMs represent a valuable option for the treatment of metastatic TC in case of isolated hepatic progression or for symptoms relief, also after the start of TKI treatment as part of a multimodal approach. The best disease control is obtained when hepatic metastatic burden is limited. These procedures are generally well tolerated; however, a cautious multidisciplinary selection of the candidates is mandatory.

## Introduction

Thyroid cancer (TC) is the most frequent endocrine malignancy. Differentiated thyroid carcinoma (DTC) arises from the epithelial follicular cells and represents the 80–90% of TCs [1]. Medullary thyroid cancer (MTC), instead, has a neuroendocrine origin and is less common (5–10% of all TCs), but it is usually more aggressive [2]. Localized TC has generally a good prognosis, while the development of distant metastases significantly increases morbidity and mortality; in case of disseminated disease, therapeutic strategies are limited [1].

Liver metastases (LMs) are quite common in advanced MTC, being found in nearly half of the patients with secondarisms [3]; on the contrary, lung and bone are the most frequent DTC metastatic sites, while LMs are quite rare [4]. Given the relative rarity of metastatic forms of TC, LMs from both DTC and MTC are, overall, relatively uncommon in clinical practice. Moreover, being frequently associated with metastatic spread in other distant organs, they are not amenable to surgery with a curative intent in most cases [5].

In the last decades, non-surgical loco-regional treatments (LRTs) including thermo-ablation techniques, such as radiofrequency ablation (RFA) or microwave ablation (MWA), and embolization techniques, such as trans-arterial embolization/chemoembolization (TAE/TACE), have been increasingly employed to treat both primary hepatic malignancy [6] and LMs from various types of cancer [7–9]. According to the ATA guidelines [10], image-guided LRTs can be performed to provide palliation or radical local tumour control in the oligo-metastatic setting; according to the specific needs of the patient, different treatment can be combined. Specifically, LRTs have been performed in both MTC and DTC patients [11–13]. The aim of this study was to describe the LRTs for LMs from TC performed at our institution and, in addition, to analyse the available English literature regarding this topic.

## Case Series

### Patients and Methods

Patients who underwent trans-arterial and/or percutaneous ablation for LMs from DTC or MTC from January 2015 to December 2020 (either as single or multiple treatments) were included.

LRTs were performed by expert interventional radiologists after multidisciplinary tumour board discussion, which involved endocrinologists, oncologists, radiation therapists, interventional radiologists, surgeons, pathologists, and nuclear medicine specialists.

Patients were considered eligible in case of first radiological detection of unresectable LM, or after the evidence of hepatic progression in absence of other metastases or with stable extrahepatic disease. If patients had been previously started a systemic therapy, LRTs were performed in case of progressive LMs when extrahepatic disease was stable. In these cases, the goal of LRTs was the achievement of a good control of liver disease allowing the prosecution of the systemic treatment.

A careful pre-evaluation of the history and the previous images of the candidates for LRTs was always performed in order to minimize the risk of complications and identify the most suitable procedure. Both severe liver insufficiency and uncorrected coagulopathy represented absolute contraindications for LRTs. LMs located at the hepatic hilum were not considered suitable for RFA/MWA, since a possible damage of main biliary ducts and vessels might occur.

Regarding the number and the size of LMs, the evaluation was performed on a case-by-case basis.

TACE was preferred in case of multiple LMs with bilobar liver involvement and adequate arterial vascularization of the lesions, while RFA/MWA was employed when a single/few lesions had to be treated. In case of persistence of vital tissue or hepatic progression of disease, a second LRT was considered. If feasible, two different procedures could be combined (e.g. RFA/MWA in conjunction with TACE in case of a large and highly vascularised LM).

All patients signed a written informed consent prior to LRT.

TACE was performed in the angio-suite under fluoroscopic guidance; femoral artery was cannulated, and selective catheterization of the hepatic artery with subsequent superselective catheterization of the feeding vessels of the tumour was performed. Drug-eluting beads (70–150-μm diameter particles) loaded with 50 mg of epirubicin were delivered intra-arterially.

Ablation was performed under ultrasound guidance: radiofrequency needle or MWA antennas were introduced into the LM to induce thermal tumour cell necrosis; the ablation area was evaluated with contrast-enhanced ultrasound at the end of the procedure.

In case of treatment with tyrosine kinase inhibitors (TKIs) with anti-VEGF (vascular endothelial growth factor) effect, the systemic treatment was temporarily interrupted in order to avoid potential bleeding.

After LRTs, contrast-enhanced computed tomography (CT) control was normally performed nearly 1 month later. Hepatic radiological response to LRT was assessed according to Response Evaluation Criteria in Solid Tumours (RECIST) criteria 1.1 [14]*.* Duration of the hepatic tumour response after the procedure was also assessed. LRT-related adverse events (AEs) in the first month after the procedure were recorded according to Common Terminology Criteria Adverse Events (CTCAE), version 5 [15]*.*

Baseline characteristics of the patients included in the analysis are summarized descriptively using median and range or number and percentages.

## Results

From January 2015 to December 2020, 17 patients with TC and LMs (8 DTC and 9 MTC) were followed at our institution. The majority of them (12 out of 17) showed progression of both LMs and extrahepatic disease; therefore, systemic therapy was preferred and no LRTs focused on LMs was performed at our centre. In one case (DTC), there was a single large LM in the absence of extrahepatic disease and the patient was referred for surgical resection.

Six hepatic LRTs were performed in the remaining 4 TC patients with LMs. As displayed in Table 1, LRTs were TACE (*n* = 3), RFA (*n* = 1), MWA (*n* = 1), or MWA combined with TACE (*n* = 1). Median age at diagnosis of TC was 37 years (range 20–65 years); all patients were female (2 MTC and 2 DTC). Median time from initial diagnosis to the detection of LM was 5.5 years (range 3.2–18.3 years); liver was the first metastatic site in 2 patients (both MTC). At the time of LRT, 2 patients (both DTC) had concurrent stable extrahepatic disease (lung and bone metastases in both patients and brain secondarisms in one case). Median time from the diagnosis of LM to the first LRT was 6 months (range 5–12 months); 2 patients were submitted to a second hepatic LRT after 8 and 11 months from the previous procedure, respectively.

**Table 1:** Main data about MTC and DTC patients treated with LRTs for LMs

*pt*	Histology (age*)	LM	Other sites of metastases	Concomitant TKI treatment	LRT	No. of treated LM	Size of largest treated LM	Response of treated LM
1	MTC (41 years)	Multiple	-	No	TACE	3	18 mm	PR
2	MTC (23 years)	Multiple	-	No	TACE	2	42 mm	PD
				Yes (vandetanib)	RFA	2	18 mm	PR
3	DTC (55 years)	Single	Bone, lung	No	MWA	1	55 mm	SD
				No	MWA + TACE	1	50 mm	SD
4	DTC (71 years)	Multiple	Bone, brain, lung	Yes (lenvatinib)	TACE	4	32 mm	SD

*DTC* differentiated thyroid cancer, *LRT* loco-regional treatment, *LM* liver metastasis, *MTC* medullary thyroid cancer, *MWA* microwave ablation, *PD* disease progression, *PR* partial response, *RFA* radiofrequency ablation, *SD* stable disease, *TACE* trans-arterial chemoembolization, *TKI* tyrosine kinase inhibitor

*Age at diagnosis of LM

A partial response was obtained in 2 cases, which lasted 18 months in one patient and is still confirmed after 20 months of follow-up in the other case. In both of them, the maximum diameter of the largest targeted lesion was 18 mm.

**Fig. 1:**
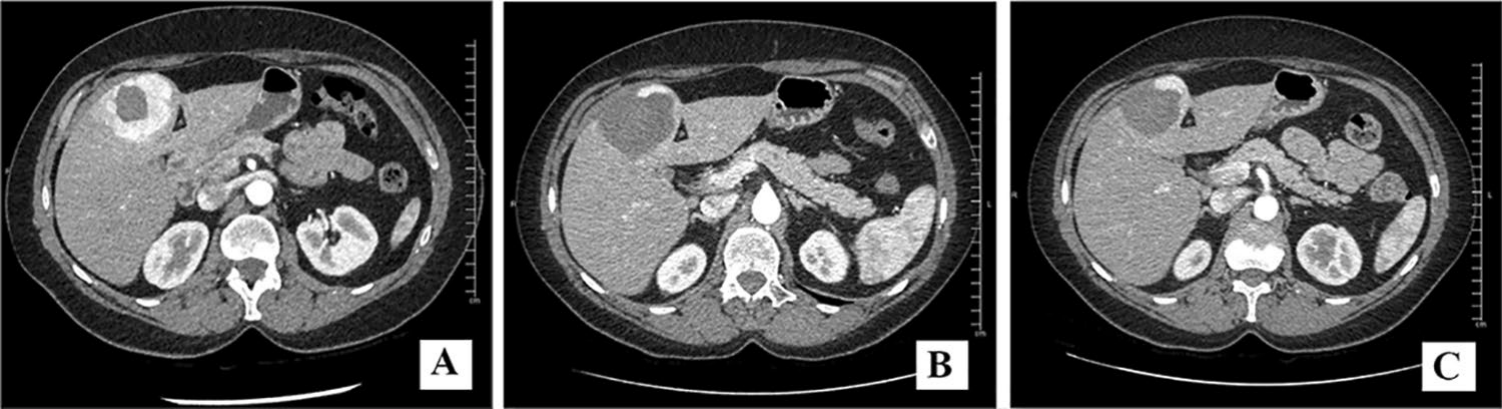
Morphological variation of subglissonian LM localized in segment IVb (patient no. 3) before the second LRT (**a**), after 1 month (**b**), and after 7 months (**c**) from the second LRT

The remaining procedures (4/6) were performed on larger lesions (range of maximum diameter 32–55 mm): in one patient (MTC with somatic M918T RET mutation), progressive disease (PD) was observed 40 days after the first LRT, with subsequent need for TKI therapy; in other cases, stable disease (SD) was achieved and maintained for more than 6 months (Fig. 1).

Acute LRT-related complications were transient and mild (G1–G2): abdominal pain in 3 patients, liver function test abnormalities in 2 cases, nausea, and toracic pain in one case each.

Two LRTs were performed in patients who were being treated with TKIs (vandetanib or lenvatinib). Systemic therapy was interrupted before and after the procedure, for a total of 11 days in one patient and 15 days in the other case; no hemorrhagic complications were recorded.

## Literature Review

Available data regarding LRTs of LMs from TC are scarce and based on small retrospective cohort studies or single case reports. A PubMed search was performed to identify all relevant English literature. Keywords (“loco-regional treatment OR therapy,” “TACE,” “RFA”) and medical subject heading terms (“thyroid cancer OR carcinoma,” “liver OR hepatic metastases”) were used to identify all potentially relevant articles. A subsequent manual crossreferencing was performed to find further pertinent studies. Table 2 summarizes current published reports about this topic [8, 11–13, 16–28].

**Table 2:** Summary of the available English articles concerning LRTs for LMs from TC

Author (year)	Type of LRT	Histology	Number of patients	Number (size of largest M)	Symptomatic response	Structural response	Reported AEs
Siperstein et al. [16]	RFA^1^	MTC	1	Single (15 mm)	NA	NA	None
Curley et al. [17]	RFA	MTC	1	NA*	NA*	NA*	NA*
Guglielmi et al. [18]	ILP	FTC	1	Single (170 mm)	Yes	PR	Fever, abdominal pain
Isozaki et al. [19]	TAE + PEI	MTC	1	Multiple (80 mm)	Yes	PR	Fever
Siperstein and Berber [20]	RFA^1^	MTC	6	NA*	NA*	NA*	NA*
Elvin et al. [21]	RFA	MTC	2	NA*	NA*	NA*	NA*
Lorenz et al. [22]	TACE	MTC	11	Multiple (size not known)	Yes (88%)	PR ^3^ (*n* = 5) SD ^3^ (*n* = 4) PD ^3^ (*n* = 1) NA (*n* = 1)	Local erythema, nausea, abdominal pain, ↑ liver enzymes (all G1) hepatic artery dissection (*n* = 1; G3) hypertensive crisis with myocardial infarction^4^ (*n* = 1; G5)
Fromigué et al. [11]	TACE	MTC	12	Multiple (median 38 mm; range 25–98)	Yes (40%)	PR (*n* = 5) SD (*n* = 5) PD (*n* = 2)	Nausea, vomiting, abdominal pain, fever, ↑ liver enzymes (all G1) post-embolization tumour necrosis with pain and fever (*n* = 1; G3)
Mazzaglia et al. [23]	RFA^1^	MTC	9	NA*	NA*	NA*	NA*
Wertenbroek et al. [12]	RFA + TAE	FTC	1	Multiple (38 mm)	-	NA	Fever
	RFA^1,2^	MTC	2	Multiple (70 mm)	Yes (100%)	Yes ^3^ (*n* = 2)	Burn wounds (n = 1)
Akyildiz et al. [24]	RFA^1^	MTC	11	NA*	NA*	NA*	NA*
Yasui et al. [25]	TACE	PTC	1	Single (size not known)	-	SD	NA
Segkos et al. [26]	MWA^1^	HTC	1	Single (21 mm)	-	CR	NA
Hughes et al. [27]	TAE	MTC	1	Multiple (size not known)	-	PR	Cholecystitis (G2)
Grozinsky-Glasberg et al. [13]	TACE	MTC	7	Multiple (median 29.5 mm; range 13–60)	Yes (100%)	PR (*n* = 7)	Nausea, vomiting, abdominal pain, fever, ↑ liver enzymes (all G1-2) hypertensive crisis (*n* = 1; G3)
Bergamini et al. [8]	TACE TAE RFA	MTC PTC	4 2	NA*	NA*	NA*	NA*
Kesim et al. [28]	TARE	PTC	1	Single (45 mm)	-	PR	NA

*AEs* adverse events, *FTC* follicular thyroid carcinoma, *HTC* Hurthle cell carcinoma, *ILP* interstitial laser photocoagulation, *MTC* medullary thyroid carcinoma, *MTS* metastases, *MWA* microwave ablation, *NA* not available, *PEI* percutaneous ethanol injection, *PD* progressive disease, *PR* partial response, *RFA* radiofrequency ablation, *RT* radiotherapy, *SD* stable disease, *TACE* transcatheter arterial chemoembolization, *TAE* transcatheter arterial embolization, *TARE* transcatheter arterial radioembolization

^1^Laparoscopic approach

^2^Laparotomic approach

^3^No RECIST-based criteria

^4^In a patient with concomitant unrecognized LMs from pheocromocytoma

*These data are not available since they refer to cohorts of patients with LMs from various tumours in which no subgroup analysis was performed

According to the retrieved articles, a total of 75 patients with TC have been treated with LRTs for LMs. MTC was the most represented histology (68/75); the majority of DTC patients had papillary thyroid cancer (4/7), while the remaining were affected by follicular (*n* = 2) or Hurthle cell (*n* = 1) carcinoma. The most commonly employed LRTs were TACE and RFA (both laparoscopic and percutaneous), while patients were less frequently treated with TAE, MWA, percutaneous ethanol injection (PEI), interstitial laser photocoagulation (ILP), and transcatheter arterial radioembolization (TARE).

In some of the published reports, TC patients with LMs have been included in larger cohorts of patients treated with LRTs for LM from other solid tumours (especially neuroendocrine tumours) or for primary hepatic malignancies [8, 17, 20, 21, 23, 24]. In these heterogeneous cohorts, data regarding the subgroups of patients with TC were not analysed separately; hence, no definitive conclusion concerning their outcomes can be retrieved. Only a small number of articles specifically focusing on TC patients have been published, and the majority of them are case reports [11–13, 16, 18, 19, 22, 25–28].

The largest studies included in this review were focused on TACE in LMs from MTC, showing that this technique is a feasible and effective procedure [11, 13, 22]. The imaging assessment of LMs after LRTs showed that PR rate varied from nearly 50% [11, 22] to 100% of the patients [13]; SD was the most common response in another significant proportion of patients (range 36–42%), while PD was less frequent. Some authors stated that radiological response seemed to be associated to the initial extent of liver involvement, with better responses in patients with less than 30% of the liver involved [11]. However, others demonstrated a beneficial effect of TACE even in case of a greater liver involvement up to 50% and with larger metastatic lesions (> 30 mm) [13]. The response was also durable, since all of these studies showed more than 12 months of ongoing response (range 14–38 months) [11, 13, 22]. TACE appears to be valuable also in controlling disease-related symptoms, since it improved both intractable diarrhoea and abdominal pain [11, 13, 22].

TACE was usually well tolerated in the majority of patients: nausea, vomiting, abdominal pain, fever (post-embolization syndrome), and elevation of liver enzymes were usually mild and transient. Few patients developed more severe AEs: hypertensive crisis in two patients [13, 22], dissection of hepatic artery, tumour necrosis syndrome, and cholecystitis in one patient each [11, 22, 27]. It is noteworthy that a greater number of AEs were recorded in patients who had been submitted repeatedly to a higher number of procedures [22]. A procedure-related death was reported in a MEN2A patient with concomitant unrecognized pheochromocytoma LMs [29].

TAE has been reported only twice in MTC, albeit with good results (PR in both patients) [19, 27].

RFA has also been employed in the treatment of LMs from MTC, but few data are available for specifically evaluating its effectiveness. Wertenbroek and colleagues reported good responses with RFA of two large lesions (70 mm), with no sign of progression after 5 years of follow-up [12].

Patients with DTC, as expected due to the rarity of this metastatic site, represent a minority of those treated with LRTs for LM: the only available detailed data are derived from few case reports [12, 18, 25, 26, 28].

Both embolization and ablative techniques have been employed for the treatment of LMs that were frequently single and large (up to 170 mm). Nonetheless, the procedures were effective in controlling the disease: two PR were achieved in 45-mm and 170-mm lesions with TARE and ILP, respectively [18, 28], and a complete response was reported in a 21-mm LM from Hürthle cell carcinoma after MWA [26]. The reported AEs were mild and self-limiting.

## Discussion

Given their rarity and the frequent association with extrahepatic advanced disease, the management of LMs from TC is challenging. In recent years, LRTs have emerged as a valuable choice in this setting, primarily for treating LMs not amenable to surgery, for symptom palliation or as ancillary procedures for other systemic therapies in case of LM progression [30]. Indeed, interventional radiology approaches for LMs from TC are also contemplated by the main international guidelines (see Table 3) [10, 30–32].

**Table 3:** Main recommendations from TC guidelines pertaining the LRTs for LMs from TC

Guideline	Disease	Recommendations
ATA 2015 [10]	DTC	Thermal ablation (RFA and cryoablation) may be considered as valid alternatives to surgery Thermal ablation should be considered prior to initiation of systemic treatment when the individual distant metastases are symptomatic or at high risk of local complications
ETA 2019 [30]	Radioiodine-refractory DTC	LRTs should be considered either alongside systemic therapies or alone in case of progression of a single lesion or multiple lesions in a single organ, with the aim of controlling symptoms, optimize disease control or delay the initiation of systemic treatments and their toxicities. When employed during systemic therapy, TKIs could be continued or interrupted temporarily for few days TACE can be applied to LMs from advanced TC, particularly when LMs are smaller than 30 mm and liver involvement is < 30%, although benefits in prolonging survival or delaying progression are yet unproven RFA can be applied to single, unresectable lesions or, in alternative, as a debulking procedure before surgical resection
ATA 2015 [31]	MTC	Treatment is indicated in patients with LMs that are large, increasing in size, or associated with symptoms such as diarrhoea or pain TACE should be considered in patients with disseminated tumors < 30 mm in size involving less than a third of the liver
ESMO 2019 [32]	DTC, MTC	If true solitary lesions are detected, they may be candidates for local ablation In MTC patients with a dominant lesion that is growing more rapidly than the background disease, local ablation (e.g. RFA) may be useful for controlling symptoms, such as diarrhea If both surgery and RFA are contraindicated, TACE might be an option

*DTC* differentiated thyroid cancer, *LMs* liver metastases, *LRTs* loco-regional treatments, *MTC* medullary thyroid cancer, *RFA* radiofrequency ablation, *TACE* trans-arterial chemoembolization, *TKI* tyrosine kinase inhibitor

There are no stringent criteria on the indications to LRTs in TC patients with LMs not only for the lack of reports in literature but also since the clinical expertise required for these techniques is not widely available. Ablation techniques should be considered in case of single or few LMs, if surgery is not indicated due to extrahepatic metastatic burden, clinical condition, or patient’s choice; conversely, a trans-arterial embolization approach should be preferred in case of diffuse liver metastatic deposits, as commonly seen in MTC [13, 24]. In addition, TACE might be employed also when an ablation procedure is contraindicated due to the LM position (e.g. near liver hilum or large bile ducts) [21].

Most of the DTC or MTC patients with liver involvement also have metastases to lymph nodes, bones, and other organs, and therefore, they require systemic therapies (e.g. TKIs, bisphosphonates). In this setting, TACE should always be carefully considered; according to our experience and the literature [11, 13, 22], it is usually well tolerated, inducing both clinical improvement and tumour response for prolonged periods of time in the majority of cases. Deterioration in liver function is the major factor determining prognosis. The best candidates for TACE should be the patients in whom the disease progression occurs mainly in the liver, irrespective of the presence of extrahepatic metastases.

Regarding embolization techniques, the good results of TAE, although in a small number of patients, leads to speculate that embolization without a chemotherapeutic agent could be a good choice for disease control, considering the added cost and potential AEs associated with TACE [12, 19, 27]. A debate focusing on the potential benefit of TACE versus TAE in patients with LMs is still ongoing [33]. Regarding TACE, no specific chemotherapeutic agent can be bound to an embolic particle for metastatic TC. The addition of doxorubicin has been shown to be associated with an increase in arterial and parenchymal necrosis and the establishment of an inflammatory response resulting in disturbances in liver metabolism [34]. Rationale for drug-eluting beads use is avoiding a peak plasma concentration of the chemotherapeutic drugs, resulting in exposure of the tumour to the therapeutic agents with less exposure of healthy liver tissue [35].

In selected cases, ablation and embolization techniques could be combined to treat the same lesions: in case of insufficient ablation with a single course of RFA, if the LM remains hypervascular, a selective cycle of TACE/TAE could be applied in order to enhance the response to a second RFA procedure [12].

Different lesion size and liver burden have been shown to influence the LRTs effectiveness. Data derived from studies carried out in patients with LMs from different types of cancers showed that LRTs seem more successful in smaller lesions and in case of limited liver tumour involvement [23, 24, 36], while a greater number of procedures might be necessary for treating larger LMs [12, 18, 19].

This finding is confirmed by our case series, since patients with lesions < 20 mm showed PR, while larger LMs remained mostly stable after LRTs. Interestingly, one of our patients (*n* = 3) with a large single LM had to undergo a second procedure after a first incomplete ablation, obtaining good results with the combined techniques.

Regarding LMs from MTC treated with TACE, a limited liver tumour involvement, but not the size of the largest LM, was associated with a better structural response [11].

Whether an early loco-regional approach might help to control the hepatic disease and delay the need of a systemic therapy is still debated; our limited data are insufficient to draw definite conclusions about this topic.

Despite the scarcity of available specific data, some lessons can be learned from the interventional radiology experience on other more common oligometastatic disease. For instance, a large series about RFA and MWA of 218 colorectal cancer LMs in 136 patients reported a 3-year local tumour progression-free survival (LTPFS) of 62%. On multivariate analysis, independent predictors of worse LTPFS were, among the others, minimal ablation margins ≤ 10 mm and LM size ≥ 2 cm [37]. Similar to those from colorectal cancer, LMs from TC could be reasonably treated with curative percutaneous ablation applying the same criteria about tumour size and ablation margins.

More in general, among factors predicting ablation site recurrence following percutaneous ablation, the most significant is the proximity of the lesion to the blood vessel [38]. Whilst a heat sink effect has been reported for RFA in the setting of LMs [39], it is unclear whether MWA is similarly affected. Technical differences between RFA and MWA including the fact that MWA reaches higher temperatures over shorter time periods suggest that MWA may be more resilient to heat sinking [40].

Although RECIST criteria are the most widely used worldwide [14], they have been found to underestimate the response of liver tumours (both primary malignancies and LMs) after LRTs [41]. This finding is related to the fact that RECIST criteria, evaluating only the shrinkage of the lesions, do not take in consideration necrosis and the decreased enhancement that characterize the response of LMs to both LRTs and new targeted therapies (e.g. TKIs). To overcome this limitation, new systems have been proposed [42, 43] that introduced the notion of “viable tumour,” the portion of the lesion that show persistent enhancement after intravenous contrast administration. New insights in the field involving also functional, volumetric, and radiomic approaches have been proposed and should be validated in future researches [41].

As observed in our series, LRTs are usually well-tolerated procedures, with temporary, mild, and manageable AEs. Side effects are generally more frequent in patients submitted to several LRTs [22]. In patients with LMs from neuroendocrine tumours, ablation is a treatment with a low complication rate, varying between 2.5 and 5.7% of all reported cases [44], and low mortality rate (0.5–1.5%) [45]*.* TACE overall morbidity and grade three-fourths complications are respectively 22.6% and 9.2% [46]. The most frequent major complications are decompensation with ascites, acute cholecystitis, acute pancreatitis, liver abscesses, and renal failure. Post-embolization syndrome appears in 20% of patients. Concomitant cardiovascular disease is a risk factor for development of complications [47].

LRTs could be employed also in the context of a multimodal strategy. In particular, ablation techniques have been shown to increase the effectiveness of radioiodine treatment [12, 18]; furthermore, endovascular procedures have been successfully employed for tumour debulking before hepatic metastasectomy [28]. Among our patients, two were treated with LRTs while on systemic therapy with TKIs, with good response and tolerance. The outcome of these cases confirms that LRTs are valuable options in combination with other treatments in advanced TC patients, especially to manage oligoprogressive metastatic disease or for symptoms relief [30].

It could be argued that the benefit from anti-angiogenic agents might be reduced by the alteration of the intratumoral vascularization by previous LRTs. On the contrary, it is also possible that the reduction of metastatic burden facilitates the cytostatic action of TKIs. No definite data are available in literature regarding this issue.

## Conclusion

LRTs for LMs represent a valuable option for the treatment of metastatic TC. The best candidates for LRTs are patients with unresectable progressive LMs with limited and stable extrahepatic disease. In case of isolated hepatic progression, LRTs can be performed also after the start of TKI treatment as part of a multimodal approach, to reduce the tumour burden and allow the prosecution of the systemic treatment. The best disease control is obtained especially when hepatic metastatic burden is limited.

These procedures are generally safe and well tolerated; however, a multidisciplinary cautious selection of the candidates for these procedures is mandatory. Prospective multicenter randomized studies, including larger number of patients with LMs, would be necessary for a better definition of the best candidates for LRTs, for treatment timing, and for evaluating its efficacy in terms of time to tumour progression and long-term survival.

## Data Availability

The data used to support the findings of this study are available from the corresponding author upon request.
